# The role of steroid injection for vocal folds lesions in professional voice users

**DOI:** 10.1186/s40463-020-00434-5

**Published:** 2020-07-20

**Authors:** Mohamed Al-Ali, Jennifer Anderson

**Affiliations:** 1 Department of Otolaryngology-HNS Surgery, University of Toronto, Toronto, ON Canada; 2 Department of Otolaryngology-Head and Neck Surgery, St. Michaels Hospital, Department of Otolaryngology-Head and Neck Surgery, University of Toronto, 30 Bond St 8CC, Toronto, ON M5B 1W8 Canada

**Keywords:** Vocal fold, Steroid, VHI-10

## Abstract

**Background:**

Benign vocal fold lesions identified in professional voice users, frequently require further treatment after failure of conservative measures. The role of vocal fold steroid injection as a treatment option for select benign lesions is the focus of this study. Steroid injection may avoid phonosurgery in some individuals thereby reducing the potential for adverse side effects associated with surgery.

**Objective:**

The purpose of the study is to review the effect of steroid injection on vocal function in professional voice users associated with a benign lesion(s) using the Voice Handicap Index-10.

**Method:**

This study is a retrospective review of patients (professional voice users) that underwent 1 or more steroid injection(s) between July 2014-December 2018. The Voice Handicap Index-10 was compared from pre to post treatment. Patients were identified using billing code data for laryngeal injection. Patient demographics (age, gender, profession), previous phonosurgery, date of steroid injection and follow up dates as well as VHI-10 scores were collected from the electronic medical record.

**Results:**

Twenty four patients were identified. The mean Voice HandicapIndex-10 score decreased from 23.5 pre injection to 17.8 post injection which represented a reduction of 24.3%. Vocal fold steroid injection procedure in our series was associated with one complication.

**Conclusion:**

Vocal fold steroid injection for benign lesions is a safe, well-tolerated procedure with an improvement in vocal function without surgical intervention. Steroid injection should be considered as a treatment option to avoid surgery in patients with select vocal fold lesions.

## Introduction

In patients who present with significant dysphonia, mucosal fold lesions are often identified during videostroboscopic examination. Benign vocal fold lesions such as vocal nodules, polyps and cysts are usually secondary to phonotrauma. These lesions are associated with chronic inflammation and variable fibrosis in the vocal fold cover and lamina propria [1, 2]. Management is multidisciplinary with medical, behavioral and potentially surgical treatment options. In professional voice users, whose livelihood may depend on their voice, small lesions which do not respond to conservative management are commonly offered surgery as these patients are more likely to have functional disability [3].

Although the risks of direct laryngoscopy (including general anaesthesia) are low, it is not without risk. Recent publications indicate that there is between 0.3–3% risk of serious complication including cardiac and airway obstruction [4]. Post operative scarring is also a concern and many professional voice users would prefer to avoid as it has been associated with irreversible mucosal wave dysfunction [5, 6]. With the trend towards non-invasive therapy and advances in both endoscopic imaging and equipment, it is possible to offer an alternate to conventional surgery with vocal fold steroid injection [7–9].

The mucosal wave of the vocal folds is generated as the cover (the epithelium and superficial lamina propria) deforms over the body (the vocal fold ligament and vocalis muscle). The underlying structure of the lamina propria consists of extracellular matrix, collagen and elastin fibers [2, 5, 6]. Benign vocal fold lesions have varying amounts of inflammatory products, collagen deposition and fibroblast activity within the mucosa and lamina propria [2]. These lesions are associated with dysphonia and in some patients with significant disability [5, 6].

Professional voice users have been defined as individuals whose profession, either wholly or partially depends on the use of voice [10]. Professional voice users typically spend 6 h per day using their voice and report higher incidence of voice symptoms and degree of disability compared to matched controls [3].

Intralesional use of corticosteroids allows a site specific administration of a high concentration therapeutic agent with a low risk of adverse systemic side effects similar to dermatological use for hypertrophic scar and keloid formation [11]. The anti-inflammatory effect of corticosteroids is posited to occur by decreased cell permeability and down-regulation of proinflammatory cytokines. Also, it has been postulated that not only does corticosteroid reduce collagen deposition but also transforming growth factor beta (TGF-beta)- induced collagen synthesis and fibroblast proliferation [11, 12].

The reported potential complications of vocal fold steroid injection (VFSI) include muscle atrophy, hemorrhage and allergic reaction [7–9] Another possible complication is the immunosuppression effect of the steroids, local or systemic [12].

## Methods

This study was approved by St Michael’s Hospital’s research ethics board (REB #19–075). Professional voice users who presented to our tertiary referral voice clinic with dysphonia were the focus of this retrospective study. Voice Handicap Index-10 (VHI-10) before and after the steroid injection for a benign lesion between July 2014–December 2018 was collected. Billing code data was used to identify all patients that underwent VFSI. Patients that received steroid injection for conditions other than dysphonia were excluded (I.E. glottic/subglottic stenosis). Professional voice users were defined as individuals whose occupation relied primarily on voice use. For example, singers, teachers or public speaking which were the most common occupations in the study group.

Of note, the use of triamcinolone acetonide (40 mg/ml) was based widely published application to treat scar. However, the suspensions can leave yellow deposits occasionally remaining visible for weeks after injection in the lamina propria. For this reason and due to the similar pharmaceutic profile, dexamethasone was used if the patient indicated a preference.

### Inclusion criteria

1. Adult patients over age 18 years of age
2. Benign appearing lesions and of small caliber (less than 50% width of true vocal fold in maximum dimension excluding granuloma)
3. Professional voice user (I.E. singers, public speaking primary occupation)
4. Optimized medical management of possible contributing comorbidities (i.e. reflux, voice use, hydration)
5. Speech therapy course completed

### Exclusion criteria

1. Leukoplakia or mucosal lesions dysplastic in appearance
2. Intolerance of flexible nasoendoscopy during initial examination (for stroboscopic evaluation)
3. Allergy to corticosteroid preparations
4. Large vocal fold lesions (phonosurgery amenable)

The following data was collected:

1. Patient demographics (age/gender/profession);
2. Diagnosis of current laryngeal pathology (polyp, nodule, prenodular oedema, scar, sulcus and granuloma)
3. History of previous vocal fold surgery
4. Date of steroid injection(s)
5. Length of follow-up
6. VHI-10 pre VFSI and post (final or single) VFSI
7. Presence of laryngo pharyngeal reflux by history /videostroboscopic findings.

The degree of dysphonia was assessed using VHI-10 which is a validated questionnaire indicating the degree of vocal disability. The response to each question was graded from 0 to 4, depending on the perceived degree of handicap with total score of 40 as most severely affected [6]. A VHI-10 score of greater or equal to 11 is abnormal [13].

## Description of VFSI

A standardized laryngeal injection flow sheet was used to document the procedure including the pathology, site of injection, amount of local anesthetic and any complications (Additional file 1). This flow sheet was adapted from the In-Office laser flow sheet that has been used in our center [14]. A separate consent was also done as per usual treatment protocol. Depending on anatomy such as small nasal space (channeled scope is 1.5 mm wider), high or narrow thyrohyoid space or difficult to palpate, and/or patient preference, one of the following two approaches were done for VFSI.

### Thyrohyoid approach

**Fig. 1:**
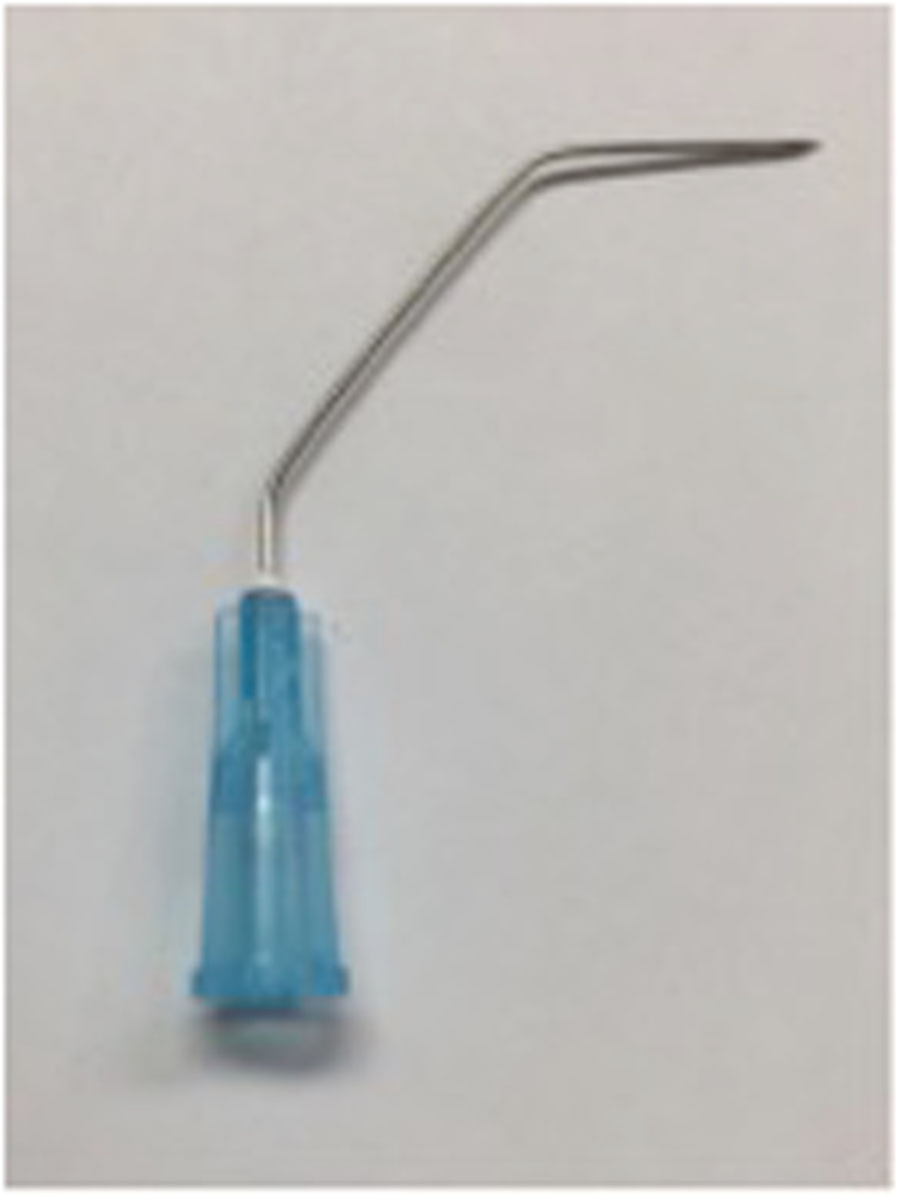
Two 45° angle bends at the hub and 1 cm from the needle tip forming a 90° angle between the syringe and tip of needle [15]

Flexible chip tip videolaryngoscopy was carried out after cottonoid packing one side of the nose with xylometazoline/4% lidocaine mixed in equal parts. Local anesthetic (1% lidocaine with 1:100,000 epinephrine) was injected at the skin at the thyroid notch and subcutaneous tissue (25 gauge needle). The assistant then advanced the videolaryngsocope (*VNL-1190STK, Pentax, JAPAN*) into position just above the vocal folds and a double bend 25G needle [15] (Fig. 1) is used to inject subcutaneously in the pre epiglottic space to just above the petiole.

Once the needle is visualized lifting the mucosa just above the vocal folds in the midline, the needle was placed through the mucosa to inject plain lidocaine (2%) onto the surface vocal folds. The needle is passed at the midline of the neck immediately above the thyroid notch and directed acutely downward once the first 45 degrees bend is at the level of the skin until the needle enters the airway in the area of the petiole. The tip of needle will tent the laryngeal mucosa before piercing to provide optimum site guidance. Approximately 1 cc of topical lidocaine is instilled in the larynx.

Alternatively, topical laryngeal anesthesia can be administered with lidocaine drip onto larynx using a working channel of the flexible laryngoscope, or dripping using Abraham cannula under flexible laryngoscope guidance, or nebulization of the lidocaine using a simple disposable nebulization device.

A 3 ml syringe filled with dexamethasone (*10 mg/ml, DIN 02387743, manufactured by OMEGA, Montreal, Canada, H3M 3A2*) or triamcinolone acetonide suspension (*40 mg/ml, DIN 01977563, manufactured by Cytex Pharmaceuticals Inc. Halifax, Canada, B3K 1 W1*) is attached to a double-bend 25G needle (1.5 in. in length was used in the same access approach as topical anesthesia). The steroid is injected in superficial lamina propria at or immediately adjacent to the site of pathology avoiding surface capillaries. The usual volume varies between 0.05–0.2 cc depending on the lesion size.

### Trans-nasal approach

Using the working channel of a flexible chip tip nasopharyngoscope (*VNL-1570STK, PENTAX, JAPAN*) with a disposable flexible needle tract (27G rigid tip, Olympus **MAJ-656) and a reusable metallic external sheath (Olympus **MAJ-655).

#### Outcome evaluation

All patients underwent history and full head and neck examination as well as videostroboscopy at the first visit. The pre VFSI VHI-10 and the last follow-up VHI-10 were collected and compared. The follow up duration was considered as the duration between the final VFSI and the last assessment with VHI-10.

#### Data analysis

Descriptive statistics were calculated and reported. Given the sample size, only the primary outcome parameter of the whole study group (VHI-10 score) before and after VFSI was analysed using paired t test with a *p* value of < .05 considered significant. Subgroup comparison was not meaningful given the small sample sizes. Confidence intervals were shown for all diagnoses subgroup data.

## Results

There were 24 patients identified who met inclusion criteria. The age range was 15–63 years with average of 38 years with 9 males and 15 females. Sixteen out of 24 are singers, 3 work in sales/marketing, one bartender, one registered nurse, and 3 were primarily public speakers. The diagnoses based on history, and videostroboscopy were pre-nodular edema/swelling (5), nodule (5), polyp (6), granuloma (2), fibrous mass/fibrosis/stiffness (4), sulcus (1) and scar (1). One of the 4 patients with vocal fold nodules also was diagnosed with a mild sulcus vocalis. The majority (92%) of patients had VFSI under local anesthesia in our clinic. Two patients under went VSFI under general anesthesia in the operating room. The number of the VFSI per patient was variable between 1 and 5 injections with 6 weeks between initial injection and a following injection.

Three patients had previous vocal fold surgery several years prior to the study. Two of these individuals underwent VFSI for scar/stiffness. One patient had a small residual polyp after microsurgery who underwent VFSI as treatment.

The average follow up was 7.6 months (range: 1-26 m, median 4.5 m). The pre procedure and the last clinic visit VHI − 10 post treatment were collected and summarized in Table 1.

**Table 1:** Subjects age, sex, pre & post VFSI VHI-10, and F/U duration

No.	Age	Sex	Pre VFSI ^a^ VHI-10 ^b^	Post VFSI VHI-10	F/U duration (months)
**1**	49	F	22	9	6
**2**	33	F	30	21	21
**3**	42	F	25	16	9
**4**	31	M	21	20	7
**5**	45	F	24	17	2
**6**	45	M	21	2	2
**7**	40	M	10	4	6
**8**	41	F	23	12	9
**9**	32	F	26	23	26
**10**	34	F	40	28	21
**11**	34	F	10	10	14
**12**	28	M	38	32	2
**13**	55	M	29	12	2
**14**	58	M	9	7	5
**15**	37	M	23	20	23
**16**	30	F	40	40	2
**17**	38	F	23	24	2
**18**	24	F	23.5	22	2
**19**	15	F	32	26	2
**20**	43	F	16	17	4
**21**	63	M	27	40	1
**22**	41	F	16	4	3
**23**	32	F	14	11	4
**24**	25	M	22	11	7

^a^ VFSI: Vocal Fold Steroid Injection

^b^ VHI-10: Voice Handicap Index-10

The mean pre VFSI VHI-10 score was 23.5 (95% CI [20.1, 26.9]), which decreased post VFSI to 17.8 (95% CI [13.7, 21.9]). Paired t test was carried out to compare the study group VHI-10 scores from pre to post VFSI which showed a *p* value of 0.04.

**Fig. 2:**
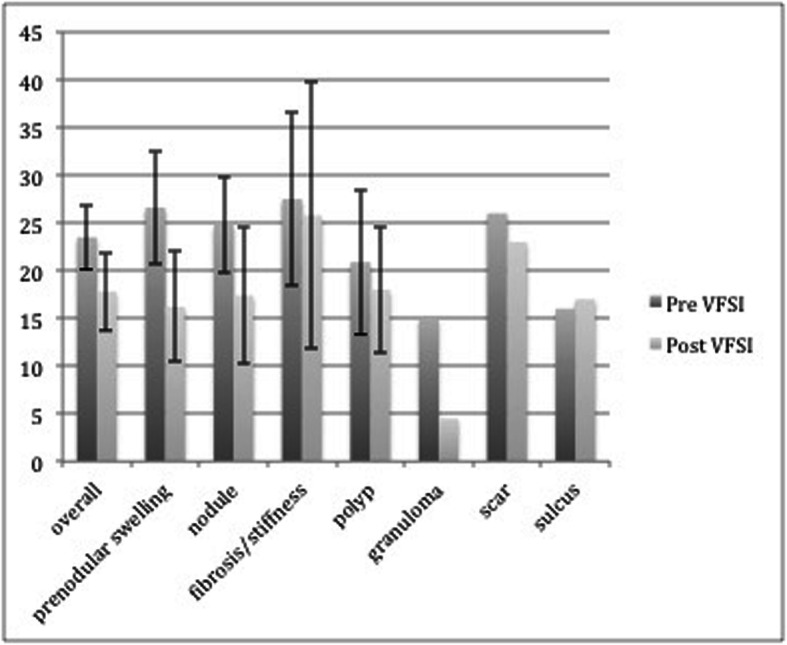
VHI-10 PRE and POST and VFSI by specific lesion diagnosis and total group

Figure 2 illustrates the VHI-10 score with confidence intervals, for the study group as a whole and by each diagnosis before and after treatment.

In general, prenodular edema and vocal nodule patients demonstrated the highest reduction in VHI-10 of 25–30% with minimal or no overlap in confidence intervals post VFSI.

However, the patients with scar/fibrosis/sulcus showed minimal or no improvement post VFSI with wider variation (wider confidence interval) post injection.

In the granuloma group (*N* = 2), the mean VHI-10 decreased from 15 in the pre VFSI to 4.5. Both patients were already on maximum medical management of known LPR for more than 6 months prior to VFSI.

A comparison was done between the VHI-10 responses to the two steroids preparations; dexamethasone 10 mg/ml and triamcinolone 40 mg/ml. Nine patients had triamcinolone 40 mg/ml and 12 patients had dexamethasone 10 mg/ml*.* Three patients were excluded in this comparison as they underwent injection with both drugs. In the triamcinolone group, the mean VHI-10 score decreased from 24.8 to 17.7 (7.1 or 28.6%). In the dexamethasone group, the average VHI-10 decreased from 21.0 to 16.8 (decreased by 4.2 or 20.1%).

There was only one complication in our series which was a vocal fold hemorrhage after VFSI. This affected her voice quality in the short term. The hemorrhage resolved and the VHI-10 decreased from 40 pre injection to 28 post VFSI.

## Discussion

The management for the benign vocal folds lesions has been primarily conservative including speech therapy and management of medical comorbidities (i.e. reflux, smoking cessation, hydration) with phonosurgery options if these measure fail. In minor/small lesions, particularly in professional voice users, weighing the potential benefit versus adverse effects of phonosurgery is a serious consideration. Vocal fold steroid injection is an option to provide an accurate, site-specific delivery of a known anti-inflammatory agent and may avoid phonosurgery in some individuals.

**Fig. 3:**
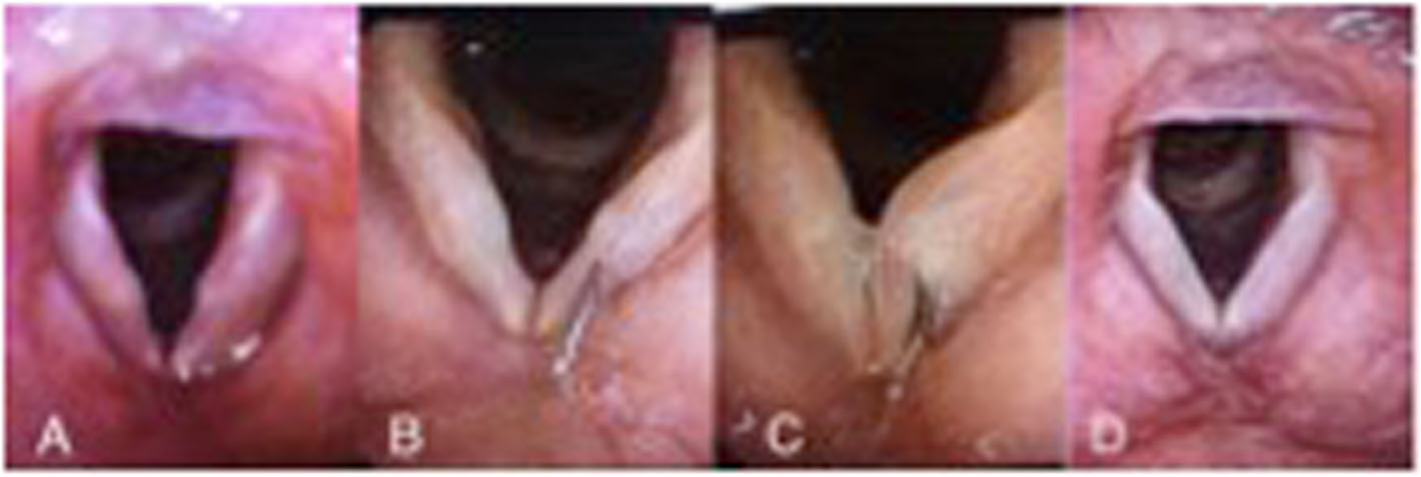
49 years old woman with prenodular edema who underwent VFSI (X2). **a**: PRE VFSI. **b** & **c**: During the VFSI. **d**: 6 months POST VFSI. VHI-10 decreased from 22 to 9

**Fig. 4:**
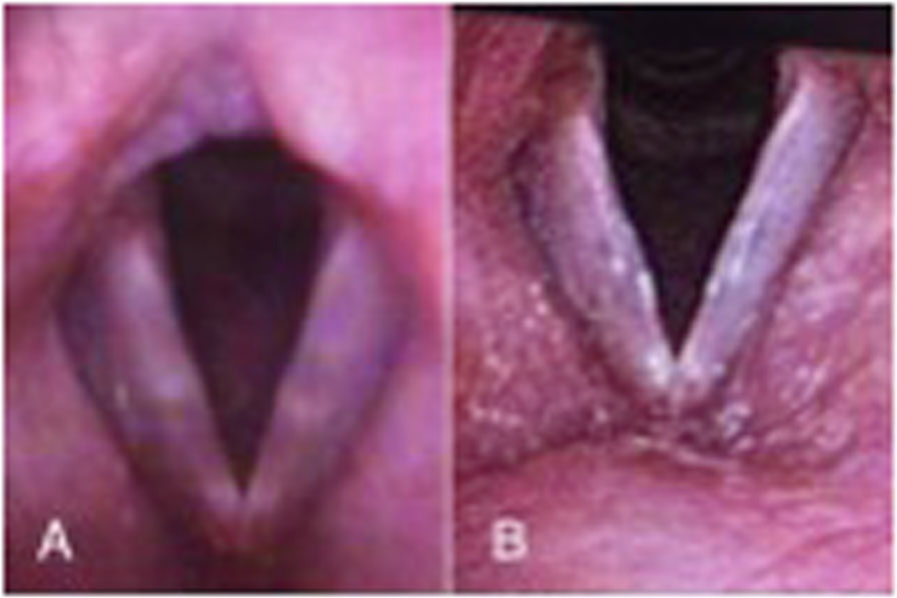
Bamboo nodules. **a**: PRE VFSI. **b**: POST VFSI. 41y/o female singer one VFSI with significant voice improvement (VHI-10 decreased from 23 to 12) in the 9 months follow up

**Fig. 5:**
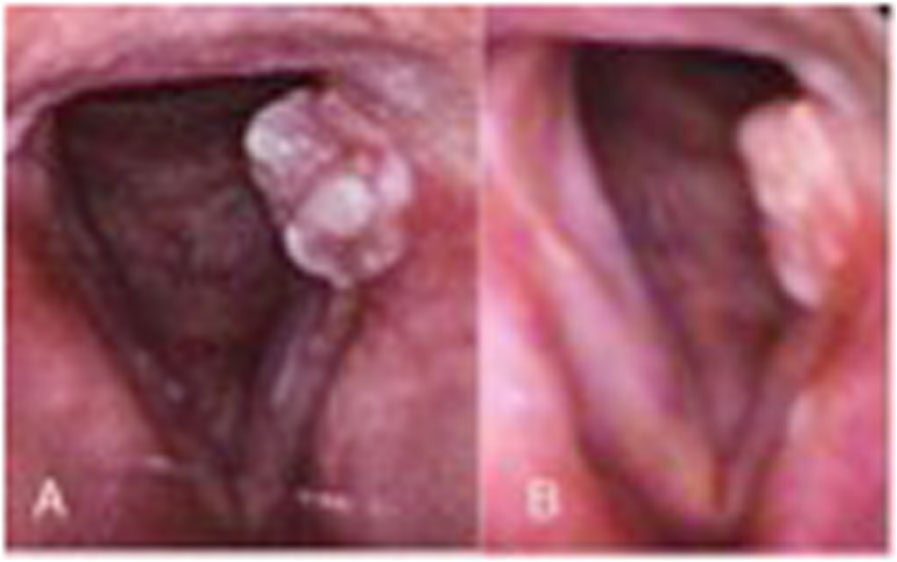
Left vocal fold granuloma. **a**: PRE VFSI. **b**: 2 months POST VFSI. This granuloma was biopsied prior to VFSI. VHI-10 decreased from 21 to 2

For example, Fig. 3 shows symptomatic prenodular edema in a female singer (patient 1 from Table 1) who had already completed both a course of speech therapy and vocal pedagogy sessions with a plateau in symptoms. Two bilateral VFSI carried out 6 weeks apart resulted in a significant reduction in VHI 10 score from 22 to 9 with improvement in stroboscopic appearance. Similarly, in patient 8 (Table 1), a singer and teacher with bamboo nodules, resistant to all conservative management responded after 3 VFSI as shown in Fig. 4. Granulomas can be very persistent and patient 6, after a year of maximal LPR treatment and behavioural voice therapy, realized a dramatic improvement after 3 VFSI over 4 months. The VHI-10 score reduced from 21 to 2 (Fig. 5).

All of these patients could have been treated with conventional microlaryngeal surgery with potentially higher risks and not necessarily better outcomes.

In our series, patients with inflammatory lesions appeared to benefit post VFSI as indicated by a reduction in VHI 10 score which was not observed in patients with scar/sulcus.

In the literature, Wang et al. [16] published a systemic review and meta-analysis describing a total of 321 patients from six reports who had VFSI for benign lesions (nodules, polyp/cyst, Reinke’s edema and scar). All six studies demonstrated subjective improvement after VFSI. Although the diagnoses included in the systematic review were different from our study group, both showed improvement in the subjective vocal function. In our study, the overall decrease in VHI-10 was 5.7 (*p* = 0.04).

As noted in other reports [7, 16], after triamcinolone acetonide injection, yellow deposits in the lamina propria can occasionally be observed which took months to resolve in a few patients. This was not found with the use of dexamethasone. Our study showed similar VHI-10 score improvement for both medications.

Wang et al. [7] showed that in a longer follow up period (median 19 months), 75% of patients showed clinical resolution of benign lesions. The cumulative failure rates (subjective symptom recurrence) at 6, 12, 18, and 24 months after VFSI were 12, 17, 24, and 32%, respectively. Similarly, Wu et al. [8] also showed a 33% recurrence rate after a single VFSI in a case series of patients with vocal fold cysts. Although our study does not have the long-term surveillance, the possible recurrence rate is important for the patient counselling and decision making.

There are limitations in our study given its retrospective nature and relatively small series of professional voice patients. Stroboscopic evaluation was not detailed in this report due to the heterogeneity of lesions identified which would make changes in appearance difficult to compare in a meaningful way. The primary outcome measure was highly relevant to voice patients - their subjective assessment of vocal function. Further subgroup statistical analysis was also not done given the small sample sizes.

Approximately half the patients were diagnosed with LPR prior to VFSI and had ongoing medical management of LPR. The compliance and response to LPR treatment was not specifically addressed in the group of VFSI patients in our study. Therefore, the role of the LPR as a confounder could not be ruled out.

In summary, vocal fold steroid injection for benign lesions is a safe, accessible treatment option with low complication rates both in our series and the literature. As a treatment options, VFSI may avoid surgery in some patients but it does not preclude microlaryngeal surgery. In our study, there was a reasonable improvement in VHI-10 scores in patients with pre nodular edema, nodules, granuloma, and to a lesser extent in vocal polyps. There was no improvement in the VHI-10 in patients with vocal fold fibrosis/ stiffness, scar, or sulcus vocalis. However, this was a very small subset of patients and it may be that a larger sample size with homogeneous diagnostic criteria may reveal usefulness in improving vocal function in this difficult to treat pathology.

Further study prospectively and multicentered would be ideal to further establish evidence of VFSI effectiveness.

## Supplementary information

**Additional file 1.** Laryngeal Injection Flowsheet.

## Data Availability

All data is stored in a secured database within the hospital according to research ethics guidelines.

## References

[1] John MM (2003). Update on the etiology, diagnosis, and treatment of vocal fold nodules, polyps, and cysts. Curr Opin Otolaryngol Head Neck Surg.

[2] Nunes RB, Behlau M, Nunes MB, Paulino JG (2013). Clinical diagnosis and histological analysis of vocal nodules and polyps. Braz J Otorhinolaryngol.

[3] Chitguppi C, Anoop R, Meher R, Rathore PK (2017). Is the voice of a professional voice user with no vocal cords lesions similar to that of non professional voice users?. J of Voice.

[4] Orosco RK, Harrusin W, Lin MD, Bhattacharyya N (2015). Safety of Adult Ambulatory Direct Laryngoscopy Revisits and Complications. JAMA Otolaryngol Head Neck Surg.

[5] Hansen JK, Thiebeault SL (2006). Current understanding and review of the literature: vocal fold scarring. J Voice.

[6] Garrett CG, Coleman J, Reinisch L. Comparative histology and vibration of the vocal folds: implications for experimental studies in microlaryngeal surgery. Laryngoscope. 2000;110:814–24. 10.1097/00005537-200005000-00011 10807360

[7] Wang C, Lai M, Cheng P (2017). Long-term surveillance following intralesional steroid injection for benign vocal fold lesions. JAMA Otolaryngol Head Neck Surg.

[8] Wu PH, Cheng PW, Lin FC, Wang CT (2018). Intralesional steroid injection as an alternative treatment for 57 patients of vocal fold mucus retention cysts. Clin Otolaryngol.

[9] Hsu Y, Liao L, Huang T, Wang C (2019). Assessment of patient outcomes after adjuvant vocal fold steroid injection for fibrosis after microlaryngeal surgery. JAMA Otolaryngol Head Neck Surg.

[10] Vilkman E (1996). Occupational risk factors and voice disorders. Logoped Phoniatr Vocol.

[11] Gauglitz GG, Korting HC, Pavicic T, Ruzicka T, Jeschke MG. Hypertrophic Scarring and Keloids: Pathologic mechanisms and Current and Emerging Treatment Strategies. Mol Med. 2011;17(1–2):113–25. 10.2119/molmed.2009.00153 PMC3022978 20927486

[12] Rhen T, Cidlowski JA (2005). Antiinflammatory action of glucocorticoids — new mechanisms for old drugs. N Engl J Med.

[13] Arffa RE, Krishna P, Gartner-Schmidt J, Rosen CA (2012). Normative values for the voice handicap Index-10. J Voice.

[14] Anderson J, Bensoussan Y, Townsley R, Kell E (2018). In-Office Endoscopic Laryngeal Laser Procedures: A Patient Safety Initiative. Otolaryngol Head Neck Surg.

[15] Achkar J, Song P, Andrus J, Franco R (2012). Double-bend needle modification for transthyrohyoid vocal fold injection. Laryngoscope.

[16] Wang CT, Liao LJ, Cheng PW, Lo WC, Lai MS (2013). Intralesional steroid injection for benign vocal fold disorders: a systematic review and meta-analysis. Laryngoscope.

