# Supplementary material for: The role of steroid injection for vocal folds lesions in professional voice users
Source: J Otolaryngol Head Neck Surg. 2020 Jul 20;49:50. doi: 10.1186/s40463-020-00434-5 (PMC7370469; doi:10.1186/s40463-020-00434-5)
Supplement: Supplementary file 1 — Additional file 1. Laryngeal Injection Flowsheet. [file 40463_2020_434_MOESM1_ESM.docx]

Patient Label

**In-Clinic Laryngeal Injection Flowsheet**

Consent

Allergies:____________________________________________

PMHX:_______________________________________________

Premedication taken by patient: __________________________

On anticoagulant?: No ___ Yes___ Drug:__________________________Date Stopped:_________________

**Procedure Preparation**

- Nasal packing by MD – Right ___ Left ______
- Lidocaine 4% - 3cc (120 mg) via nebulizer __________mg
- Lidodan endotracheal spray(12mg/spray) - _______ spray(s) __________mg
- Lidocaine 2 % / 4% topical via spray catheter __________mg
- Lidocaine 2% (2cc/40 mg) transtracheal injection(**MD only**) ___________mg

**Total ___________ mg**

**Injection**

Pentax ____ Olympus ______ Storz_________

Serial # __________________________ Injector needle sticker

**
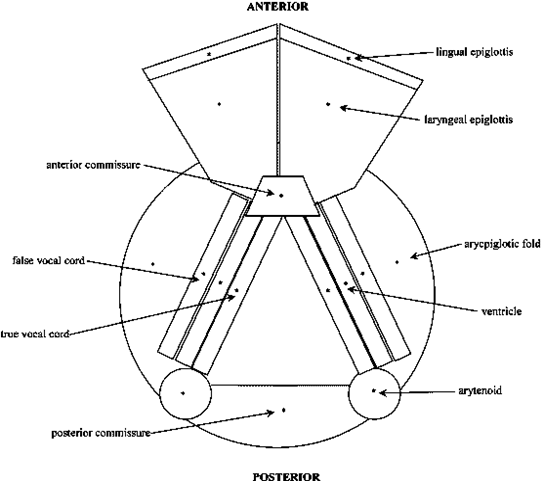
**

**Transcutaneous Injection**

Triamcinolone 10 mg/ml ______________mg

Triamcinolone 40 mg/ml _____________ mg

Dexamethasone 4mg/ml ------------------- mg

Dexamethasone 10 mg/ml ____________mg

Radiesse __________________________ml

Restylane __________________________ml

Botox ____________________________units
 Prolaryn Plus _______________________cc

**SMH ENT Clinic – Revised January 2020**
